# Prevalence and Changes in Preexisting Diabetes and Gestational Diabetes Among Women Who Had a Live Birth — United States, 2012–2016

**DOI:** 10.15585/mmwr.mm6743a2

**Published:** 2018-11-02

**Authors:** Nicholas P. Deputy, Shin Y. Kim, Elizabeth J. Conrey, Kai McKeever Bullard

**Affiliations:** ^1^Division of Reproductive Health, National Center for Chronic Disease Prevention and Health Promotion, CDC; ^2^Oak Ridge Institute for Science and Education, Oak Ridge, Tennessee; ^3^Ohio Department of Health; ^4^Division of Diabetes Translation, National Center for Chronic Disease Prevention and Health Promotion, CDC.

Diabetes during pregnancy increases the risk for adverse maternal and infant health outcomes. Type 1 or type 2 diabetes diagnosed before pregnancy (preexisting diabetes) increases infants’ risk for congenital anomalies, stillbirth, and being large for gestational age (*1*). Diabetes that develops and is diagnosed during the second half of pregnancy (gestational diabetes) increases infants’ risk for being large for gestational age (*1*) and might increase the risk for childhood obesity (*2*); for mothers, gestational diabetes increases the risk for future type 2 diabetes (*3*). In the United States, prevalence of both preexisting and gestational diabetes increased from 2000 to 2010 (*4*,*5*). Recent state-specific trends have not been reported; therefore, CDC analyzed 2012–2016 National Vital Statistics System (NVSS) birth data. In 2016, the crude national prevalence of preexisting diabetes among women with live births was 0.9%, and prevalence of gestational diabetes was 6.0%. Among 40 jurisdictions with continuously available data from 2012 through 2016, the age- and race/ethnicity-standardized prevalence of preexisting diabetes was stable at 0.8% and increased slightly from 5.2% to 5.6% for gestational diabetes. Preconception care and lifestyle interventions before, during, and after pregnancy might provide opportunities to control, prevent, or mitigate health risks associated with diabetes during pregnancy.

NVSS collects data for all live births in 50 states, New York City,* and District of Columbia (DC).^†^ The U.S. Standard Certificate of Live Birth (birth certificate) uniformly documents birth-related information across jurisdictions and was revised in 2003 to include distinct fields for preexisting and gestational diabetes; the National Center for Health Statistics recommends information about these conditions be collected from prenatal care records, labor and delivery forms, or delivery records.^§^ The birth certificate also includes information on maternal characteristics, which might be self-reported or collected from medical records.^¶^ The revised birth certificate was implemented in 40 jurisdictions as of 2012** (representing 86.3% of live births to U.S. residents) and in all jurisdictions as of January 2016.

The national prevalences of preexisting and gestational diabetes were calculated for U.S. resident mothers who had a live birth in 2016. Crude prevalences were calculated overall and by selected maternal characteristics among women with complete information for each particular characteristic^††^; chi-square tests were used to evaluate differences by characteristic. To examine changes in prevalence of preexisting and gestational diabetes, jurisdiction-specific prevalences were calculated for U.S. resident mothers with a live birth during 2012–2016 and who were residing in jurisdictions that adopted the revised birth certificate by January 1 of the year in which they gave birth; women with missing data on diabetes status (<1%) were excluded from this portion of the analysis. Jurisdiction-specific prevalences were calculated for each year after directly standardizing to the distribution of age and race/ethnicity of U.S. resident mothers with live births in 2012 because these characteristics vary by jurisdiction and are nonmodifiable determinants of diabetes. For 40 jurisdictions with data available from 2012 to 2016 (n = 17,050,514 women; 86% of U.S. resident women with live births during 2012–2016), differences in standardized prevalences between 2012 and 2016 were calculated for each jurisdiction and for all jurisdictions combined; differences were assumed to be independent and were evaluated using the z-statistic. P-values <0.05 were considered statistically significant.

In 2016, the crude national prevalences of preexisting and gestational diabetes were 0.9% and 6.0%, respectively (Table 1); prevalence varied by all characteristics examined (p<0.05). For example, by race/ethnicity, the prevalence of preexisting diabetes was highest among American Indian/Alaska Native women (2.1%) and Native Hawaiian/Pacific Islander women (1.8%), and the prevalence of gestational diabetes was highest among non-Hispanic Asian women (11.1%). The prevalences of both preexisting and gestational diabetes varied by prepregnancy body mass index (BMI): among underweight women, the prevalences of preexisting diabetes and gestational diabetes were 0.3% and 2.9%, respectively; whereas among women with class III obesity, the respective prevalences were 3.2% and 13.9%.

**TABLE 1:** Unadjusted prevalences of preexisting diabetes and gestational diabetes among women with a live birth, by selected maternal characteristics — United States, 2016

Characteristic*	No.^†^	% Preexisting diabetes	% Gestational diabetes
**Total**	**3,942,094**	**0.9**	**6.0**
**Age group (yrs)**			
<20	211,827	0.4	1.9
20–24	803,153	0.5	3.3
25–29	1,148,057	0.7	5.1
30–34	1,110,010	1.0	7.0
35–39	546,995	1.4	9.6
≥40	122,052	2.1	12.8
**Race and Hispanic origin** ^§^			
White, non-Hispanic	2,054,437	0.7	5.3
Black, non-Hispanic	558,044	1.2	4.8
Asian, non-Hispanic	254,326	0.9	11.1
Hispanic	917,822	1.0	6.6
American Indian/Alaska Native	31,375	2.1	9.2
Native Hawaiian/Pacific Islander	9,337	1.8	8.4
More than one race	80,836	0.9	5.8
**Nativity**			
U.S.-born	3,024,356	0.8	5.2
Not U.S.-born	909,638	0.9	8.4
**Education**			
Less than high school	537,990	1.1	6.2
High school graduate	978,917	0.9	5.5
Some college	1,128,682	1.0	6.2
College graduate	784,655	0.6	5.9
More than college	460,768	0.6	6.0
**Payment source for delivery**			
Medicaid	1,668,864	1.0	5.9
Private	1,936,143	0.8	6.2
Other^¶^	313,437	0.7	5.1
**Trimester entry into prenatal care**			
First	2,955,378	0.9	6.2
Second	639,593	0.8	5.6
Third or none	235,409	0.7	4.6
**Parity**			
Nulliparous	1,498,458	0.8	5.2
Primiparous	1,263,445	0.8	5.9
Multiparous	1,165,053	1.0	7.1
Prepregnancy body mass index**			
Underweight	134,392	0.3	2.9
Normal weight	1,699,751	0.4	3.6
Overweight	997,977	0.8	6.1
Obesity Class I	548,092	1.3	8.8
Obesity Class II	266,105	2.0	11.2
Obesity Class III	187,689	3.2	13.9

* Statistically significant (p<0.05) differences in the distribution of preexisting diabetes, gestational diabetes (or no diabetic conditions) were observed by all maternal characteristics.

^†^ The number of women within a characteristic group (e.g., age group) might not sum to the total number of women because of missing information.

^§^ Race and Hispanic origin are reported separately on the birth certificate. Women reporting Hispanic origin were categorized as Hispanic regardless of their race. Categories represent single-race reporting (i.e., mothers reported only one race); mothers reporting more than one race were categorized as “More than one race.”

^¶^ Includes insurance provided by TRICARE or the Indian Health Service.

** Prepregnancy body mass index (BMI; kg/m^2^) classified as underweight (BMI <18.5), normal weight (BMI 18.5–24.9), overweight (BMI 25.0–29.9), obesity class I (BMI 30.0–34.9), obesity class II (35.0–39.9), and obesity class III (BMI ≥40.0).

After standardizing for age and race/ethnicity, the 2016 prevalence of preexisting diabetes ranged from 0.5% in California to 1.7% in West Virginia (Table 2) (Figure); prevalence of gestational diabetes ranged from 3.4% in DC to 9.2% in South Dakota (Table 2) (Figure). From 2012 to 2016, among the 40 jurisdictions with continuously available data, the standardized prevalence of preexisting diabetes was stable at 0.8% (Table 2). Statistically significant increases in the prevalence of preexisting diabetes were observed in eight jurisdictions (range = 0.1% [California] to 0.3% [Georgia]); a significant decrease was observed only for Oklahoma (0.4%). From 2012 to 2016, the standardized prevalence of gestational diabetes increased from 5.2% to 5.6%. Statistically significant increases in the prevalence of gestational diabetes were observed in 22 jurisdictions (range = 0.3% [Illinois] to 3.2% [South Dakota]); significant decreases were observed in six jurisdictions (range = 0.4% [Massachusetts] to 1.9% [New Hampshire]).

**TABLE 2:** Standardized* prevalence of preexisting and gestational diabetes among women with a live birth, by jurisdiction, year, and percentage point change — United States, 2012–2016

Jurisdiction	Percentage of women with preexisting diabetes						Percentage of women with gestational diabetes					
	2012	2013	2014	2015	2016	Percentage-point difference, 2012 to 2016 (95% CI)^†^	2012	2013	2014	2015	2016	Percentage-point difference, 2012 to 2016 (95%CI)^†^
Alabama	—^§^	—^§^	1.1	1.1	1.1	—^§^	—^§^	—^§^	4.6	4.8	5.3	—^§^
Alaska	—^§^	0.6	1.0	1.0	0.9	—^§^	—^§^	7.2	6.9	6.7	8.3	—^§^
Arizona	—^§^	—^§^	0.8	0.8	0.8	—^§^	—^§^	—^§^	5.7	6.9	6.9	—^§^
Arkansas	—^§^	—^§^	1.0	1.0	1.0	—^§^	—^§^	—^§^	5.2	5.4	5.6	—^§^
California	0.4	0.4	0.4	0.4	0.5	0.1 (0.0 to 0.1)^†^	4.2	4.4	4.7	4.6	4.6	0.4 (0.4 to 0.5)^†^
Colorado	0.7	0.8	0.7	0.6	0.7	0.0 (-0.1 to 0.1)	4.2	4.4	4.2	4.2	4.3	0.1 (-0.1 to 0.4)
Connecticut	—^§^	—^§^	—^§^	—^§^	0.8	—^§^	—^§^	—^§^	—^§^	—^§^	5.7	—^§^
Delaware	1.0	0.9	0.8	0.8	0.9	-0.2 (-0.4 to 0.1)	7.5	6.9	7.9	7.2	7.2	-0.3 (-1.0 to 0.5)
District of Columbia	0.6	0.8	0.8	0.5	0.8	0.1 (-0.2 to 0.4)	2.9	3.0	2.8	2.5	3.4	0.5 (-0.3 to 1.3)
Florida	0.8	0.8	0.7	0.7	0.8	-0.1 (-0.1 to 0.0)	5.0	4.6	4.4	4.3	4.4	-0.5 (-0.7 to -0.4)^†^
Georgia	0.7	0.8	0.9	1.0	0.9	0.3 (0.2 to 0.4)^†^	4.0	4.1	3.8	3.8	4.7	0.7 (0.5 to 0.8)^†^
Hawaii	—^§^	—^§^	0.6	0.5	0.5	—^§^	—^§^	—^§^	3.3	4.5	3.8	—^§^
Idaho	0.7	0.7	0.7	0.7	1.0	0.3 (-0.0 to 0.6)	5.7	6.3	5.6	6.6	5.8	0.1 (-0.7 to 0.9)
Illinois	0.8	0.8	0.7	0.8	0.9	0.1 (0.0 to 0.1)	5.9	6.2	6.0	6.3	6.3	0.3 (0.2 to 0.5)
Indiana	1.1	1.0	1.0	1.0	1.0	-0.0 (-0.2 to 0.1)	6.7	6.2	6.1	6.2	6.9	0.1 (-0.2 to 0.4)
Iowa	1.0	1.0	1.2	1.2	1.3	0.2 (0.0 to 0.4)^†^	7.2	8.0	7.8	8.3	8.4	1.1 (0.6 to 1.6)^†^
Kansas	0.8	0.9	0.9	0.8	0.8	-0.0 (-0.2 to 0.1)	5.8	5.7	6.0	6.0	6.4	0.5 (0.2 to 0.9)^†^
Kentucky	1.1	1.0	1.1	1.1	1.1	0.1 (-0.1 to 0.2)	6.2	5.9	6.1	6.0	6.4	0.2 (-0.2 to.6)
Louisiana	0.8	0.9	0.9	1.0	1.0	0.2 (0.1 to 0.3)^†^	5.0	6.1	6.0	5.9	5.9	0.9 (0.5 to 1.2)^†^
Maine	—^§^	—^§^	1.0	1.0	0.9	—^§^	—^§^	—^§^	6.5	6.0	6.2	—^§^
Maryland	0.8	0.8	0.8	0.8	0.8	0.0 (-0.1 to 0.1)	4.8	5.0	5.6	5.9	5.9	1.1 (0.8 to 1.3)^†^
Massachusetts	0.7	0.8	0.7	0.8	0.8	0.1 (-0.0 to 0.2)	5.2	4.8	4.8	5.2	4.8	-0.4 (-0.6 to -0.1)^†^
Michigan	0.9	0.8	0.8	0.8	0.9	0.1 (-0.0 to 0.2)	6.2	5.4	5.5	5.4	5.5	-0.7 (-1.0 to -0.5)^†^
Minnesota	1.1	0.9	1.0	1.0	0.9	-0.1 (-0.3 to 0.0)	7.0	7.1	6.7	6.7	7.1	0.1 (-0.2 to 0.5)
Mississippi	—^§^	0.8	1.0	0.8	0.8	—^§^	—^§^	4.9	4.5	4.3	4.3	—^§^
Missouri	0.8	0.8	0.8	1.0	0.8	0.0 (-0.1 to 0.2)	6.0	5.8	5.9	6.2	6.8	0.8 (0.4 to 1.1)^†^
Montana	0.7	0.5	1.0	0.9	0.9	0.2 (-0.3 to 0.7)	2.8	4.0	4.6	5.2	4.7	1.8 (0.9 to 2.8)^†^
Nebraska	0.9	1.0	1.0	1.0	1.0	0.1 (-0.1 to 0.3)	5.8	6.4	5.7	6.0	6.5	0.7 (0.3 to 1.2)^†^
Nevada	0.9	0.8	0.9	0.9	1.0	0.1 (-0.1 to 0.2)	5.1	5.6	5.5	5.4	5.9	0.8 (0.4 to 1.1)^†^
New Hampshire	0.7	0.8	0.8	0.7	0.7	-0.0 (-0.3 to 0.3)	7.3	6.6	6.9	5.2	5.5	-1.9 (-3.0 to -0.7)^†^
New Jersey	^—§^	—^§^	—^§^	—^§^	0.8	—^§^	—^§^	—^§^	—^§^	—^§^	5.9	—^§^
New Mexico	0.8	0.9	0.9	0.9	0.8	-0.0 (-0.3 to 0.2)	3.4	3.5	4.3	4.4	4.7	1.4 (0.9 to 1.9)^†^
New York	0.7	0.7	0.8	0.7	0.8	0.2 (0.1 to 0.3)^†^	5.2	5.3	5.7	6.0	6.3	1.1 (1.0 to 1.3)^†^
New York City^¶^	0.5	0.5	0.5	0.5	0.5	0.0 (-0.0 to 0.1)	3.9	3.7	4.3	5.2	5.9	2.0 (1.8 to 2.2)^†^
North Carolina	0.8	0.8	0.8	0.9	1.0	0.2 (0.1 to 0.3)^†^	5.9	5.8	5.6	5.5	5.8	-0.0 (-0.2 to 0.2)
North Dakota	0.8	0.7	0.7	1.2	0.8	-0.0 (-0.4 to 0.4)	5.2	5.6	5.3	6.5	6.2	1.0 (-0.0 to 2.1)
Ohio	1.0	1.0	1.0	1.0	1.1	0.1 (-0.0 to 0.2)	7.6	7.8	7.67	8.0	8.2	0.6 (0.3 to 0.9)^†^
Oklahoma	1.2	0.8	0.9	0.9	0.9	-0.4 (-0.5 to -0.2)^†^	4.3	4.5	4.7	4.8	5.1	0.9 (0.5 to 1.2)^†^
Oregon	1.0	0.9	1.0	0.8	1.0	0.1 (-0.1 to 0.2)	7.5	8.0	8.1	8.0	8.1	0.6 (0.1 to 1.0)^†^
Pennsylvania	0.8	0.8	0.8	0.8	0.8	-0.0 (-0.1 to 0.0)	5.5	5.4	5.6	5.5	5.5	0.1 (-0.2 to 0.3)
Rhode Island	—^§^	—^§^	—^§^	0.7	0.8	—^§^	—^§^	—^§^	—^§^	6.7	6.1	—^§^
South Carolina	1.0	1.1	0.9	0.9	1.0	0.0 (-0.1 to 0.2)	5.9	6.5	7.3	7.0	7.1	1.1 (0.8 to 1.5)^†^
South Dakota	0.7	0.8	0.8	0.7	0.7	0.1 (-0.3 to 0.4)	6.1	7.1	8.5	8.4	9.2	3.2 (2.1 to 4.3)^†^
Tennessee	1.0	1.1	1.3	1.2	1.2	0.2 (0.1 to 0.3)^†^	7.0	6.7	6.1	6.2	6.1	-0.9 (-1.2 to -0.6)^†^
Texas	0.7	0.7	0.7	0.7	0.6	-0.0 (-0.1 to 0.0)	4.2	4.0	4.5	4.5	4.6	0.4 (0.3 to 0.5)^†^
Utah	0.7	0.7	0.7	0.9	0.7	0.0 (-0.1 to 0.2)	4.8	5.0	5.6	6.4	6.4	1.6 (1.1 to 2.1)^†^
Vermont	0.6	0.8	1.1	1.2	1.0	0.3 (-0.3 to 1.0)	4.4	6.3	4.2	4.0	4.3	-0.1 (-1.5 to 1.2)
Virginia	—^§^	0.6	0.5	0.7	0.7	—^§^	—^§^	4.2	4.8	5.1	5.3	—^§^
Washington	0.8	0.8	0.8	0.9	0.9	0.1 (0.1 to 0.2)^†^	6.7	6.7	7.0	7.6	7.8	1.0 (0.8 to 1.3)^†^
West Virginia	—^§^	—^§^	2.0	1.5	1.7	—^§^	—^§^	—^§^	6.7	7.1	7.2	—^§^
Wisconsin	1.1	1.2	1.0	1.0	1.1	0.1 (-0.1 to 0.2)	7.0	7.1	7.0	6.9	6.6	-0.4 (-0.7 to -0.1)^†^
Wyoming	0.9	0.9	0.8	1.0	0.6	-0.3 (-1.0 to 0.3)	3.3	3.3	3.7	4.6	3.8	0.5 (-0.4 to 1.3)
40 jurisdictions with data during 2012–2016**	**0.8**	**0.8**	**0.8**	**0.8**	**0.8**	**0.1 (0.0 to 0.1)^†^**	**5.2**	**5.2**	**5.4**	**5.5**	**5.6**	**0.4 (0.4 to 0.5)^†^**

**Abbreviation:** CI = confidence interval.

* Standardized to the age and race/ethnicity distribution of U.S. resident mothers delivering in 2012.

^†^ Statistically significant (p<0.05) difference from 2012 to 2016.

^§^ A dash indicates revised birth certificates were not available by January 1 of that year for that jurisdiction.

^¶^ Natality data from New York City are reported separately and are not included in New York state estimates.

** Among the 40 jurisdictions with data during 2012–2016, the sample sizes were 3,391,723 (2012); 3,378,197 (2013); 3,435,616 (2014); 3,434,815 (2015); and 3,410,163 (2016).

**FIGURE:**
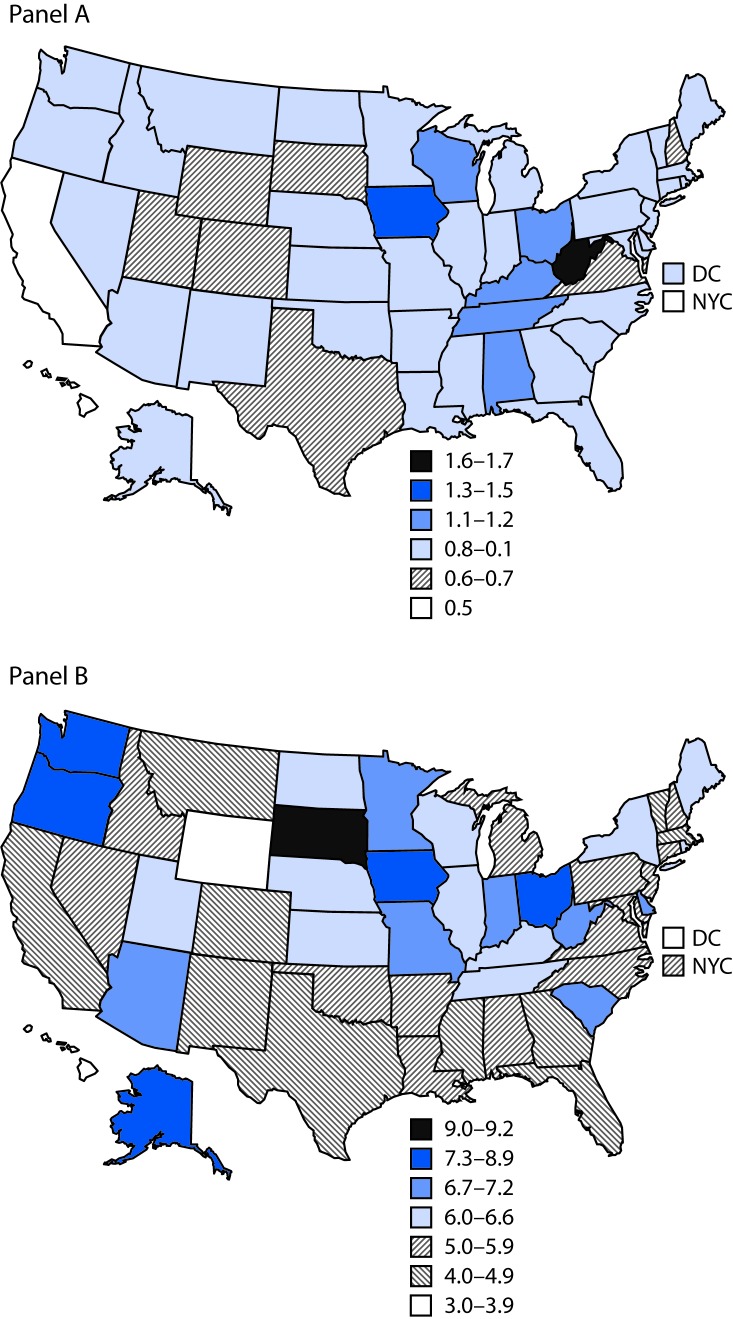
Standardized* prevalence of preexisting (panel A) and gestational (panel B) diabetes among women who had a live birth — United States, 2016 **Abbreviations:** DC = District of Columbia; NYC = New York City. * Standardized to age and race/ethnicity distribution of U.S. resident mothers delivering in 2012.

## Discussion

In 2016, the crude national prevalences of preexisting and gestational diabetes were 0.9% and 6.0%, respectively.^§§^ From 2012 to 2016 among 40 jurisdictions with continuously available data, the age- and race/ethnicity-standardized prevalence of preexisting diabetes remained stable (<0.1 percentage point change), and the prevalence of gestational diabetes increased by 0.4 percentage point. Changes in preexisting and gestational diabetes reported here extend findings from two studies using hospital discharge data from 19 states; these studies found the age-adjusted prevalence of preexisting diabetes increased from 0.7% to 0.9% from 2000 to 2010, and the prevalence of gestational diabetes increased from 3.7% to 5.8% (*4*,*5*). Observed increases in the prevalence of preexisting and gestational diabetes might reflect, in part, recent increases in the prevalence of prepregnancy obesity.^¶¶^Estimates of preexisting diabetes may be leveling off compared to what has been seen in recent years. The high prevalence of gestational diabetes in Asian women is consistent with previous literature (*5*). Preconception care and lifestyle interventions before, during, and after pregnancy might provide opportunities to control, prevent, or mitigate health risks associated with diabetes during pregnancy.

Preconception care refers to health care before pregnancy that optimizes a woman’s health and pregnancy-related outcomes, should a pregnancy occur.*** Preconception care provides an opportunity to reinforce the importance of diabetes management among reproductive-aged women with type 1 or type 2 diabetes and might reduce adverse pregnancy outcomes by improving glycemic control before critical developmental stages of the fetus early in pregnancy (*6*). Because prepregnancy overweight and obesity are strongly associated with developing gestational diabetes, preconception care offers an opportunity to provide all women with recommended BMI screening and to refer women with obesity to intensive multicomponent behavioral interventions.^†††^

Gestational diabetes strongly predicts the development of future type 2 diabetes (*3*). Women with gestational diabetes are recommended to receive testing for type 2 diabetes 4–12 weeks postpartum and, if diabetes is detected, referred for follow-up care; lifelong monitoring is recommended for women with normal results.^§§§^ Although national estimates of postpartum diabetes testing are unavailable, some studies report suboptimal testing rates (*7*), suggesting missed opportunities to provide health care for women with diabetes and those at risk for developing diabetes.

Structured lifestyle change programs that promote a healthy diet and increase physical activity, such as CDC-recognized programs coordinated through the National Diabetes Prevention Program, reduce the risk for type 2 diabetes in nonpregnant populations at high risk.^¶¶¶^ During the first half of pregnancy, lifestyle interventions might reduce the risk for developing gestational diabetes; however, additional research is needed to understand the most successful intervention designs (*8*). Among women who had gestational diabetes but did not develop type 2 diabetes after pregnancy, postpartum lifestyle interventions have been found to reduce postpartum weight retention and improve markers of insulin resistance (*9*). Importantly, postpartum mothers face unique barriers to engaging in lifestyle interventions, including childcare responsibilities and time constraints (*9*).

The findings in this report are subject to at least five limitations. First, prevalences of preexisting and gestational diabetes might be underestimated because of underreporting or incomplete birth certificate information, the degree of which might vary by jurisdiction, or because this study was limited to live births; studies indicate sensitivity of identifying preexisting diabetes from birth certificates ranges from 47%–52%, whereas sensitivity for identifying gestational diabetes ranges from 46%–83% (*10*). Second, recommendations for gestational diabetes screening changed in 2014, and diagnostic criteria might vary by individual practice; consequently, differences in prevalence over time or by jurisdiction might reflect variations in screening or diagnostic practices. Third, analyses examining changes over time were limited to 40 jurisdictions with available data and, as a result, do not represent the entire U.S. population of women giving birth. Fourth, differences in standardized prevalences between the two times do not necessarily imply a steady rate of change during the entire period, which might not reflect actual variation observed. Finally, some statistically significant findings might be driven by large sample sizes and might not reflect a meaningful change.

In 2016, the national prevalences of preexisting and of gestational diabetes were 0.9% and 6.0%, respectively, and prevalences of both conditions increased slightly from 2012 to 2016; notably, standardized prevalences and changes over time varied by jurisdiction. Preconception care and lifestyle interventions before, during, and after pregnancy might prevent, control, or mitigate risks associated with diabetes during pregnancy.

**Summary**

**What is already known about this topic?**

Diabetes diagnosed before (preexisting diabetes) and during (gestational diabetes) pregnancy increases the risk for adverse infant and maternal health outcomes. Recent prevalence and trend estimates for these conditions have not been reported.

**What is added by this report?**

In 2016, the national prevalences of preexisting and gestational diabetes were 0.9% and 6.0%, respectively. Among 40 jurisdictions, the age- and race/ethnicity-standardized preexisting diabetes prevalence was stable at 0.8%, and the gestational diabetes prevalence increased from 5.2% to 5.6%.

**What are the implications for public health practice?**

Changes in preexisting and gestational diabetes suggest strategies before, during, and after pregnancy are needed to prevent, control, or mitigate risks associated with these conditions.

## Footnotes

* New York City birth data are reported separately from New York state birth data and are not included in New York state estimates.

† NVSS also collects information for U.S. territories (American Samoa, Guam, Northern Mariana Islands, Puerto Rico, and U.S. Virgin Islands); however, data for these areas are not included in this report.

§ The National Center for Health Statistics guidance for completing the 2003 revision of the U.S. Standard Certificate of Live Birth recommends information on preexisting diabetes and gestational diabetes be collected from the following sources, in order: the prenatal care record, labor and delivery nursing admission triage form, admission history and physical form, or delivery record. https://www.cdc.gov/nchs/data/dvs/GuidetoCompleteFacilityWks.pdf.

¶ Most maternal demographic information is collected by self-report, whereas most maternal medical and health data are collected from medical records. Additional information is available from the National Center for Health Statistics guidance for completing the 2003 revision of the U.S. Standard Certificate of Live Birth (https://www.cdc.gov/nchs/data/dvs/birth_edit_specifications.pdf); however, guidance might vary by jurisdiction because recommendations may be modified to better suit each jurisdiction’s needs.

** The 40 jurisdictions that adopted the revised birth certificate by 2012 are California, Colorado, Delaware, District of Columbia, Florida, Georgia, Idaho, Illinois, Indiana, Iowa, Kansas, Kentucky, Louisiana, Maryland, Massachusetts, Michigan, Minnesota, Missouri, Montana, Nebraska, Nevada, New Hampshire, New Mexico, New York, New York City, North Carolina, North Dakota, Ohio, Oklahoma, Oregon, Pennsylvania, South Carolina, South Dakota, Tennessee, Texas, Utah, Vermont, Washington, Wisconsin, and Wyoming.

†† Overall, <1% of live births had missing information on diabetic status. For maternal characteristics, among women with complete information on diabetic status, missing data ranged from <1% for nativity to 2.8% for trimester of entry into prenatal care.

§§ Findings are consistent with prevalence estimates reported by the National Center for Health Statistics (https://www.cdc.gov/nchs/data/nvsr/nvsr67/nvsr67_01.pdf).

¶¶ https://www.cdc.gov/mmwr/volumes/66/wr/mm665152a3.htm.

*** https://www.cdc.gov/mmwr/preview/mmwrhtml/rr5506a1.htm.

††† The U.S. Preventive Services Task Force recommends screening all adults for obesity and referrals for patients with obesity to intensive, multicomponent behavioral interventions (https://www.uspreventiveservicestaskforce.org/Page/Document/RecommendationStatementFinal/obesity-in-adults-screening-and-management).

§§§ Updated American College of Obstetrics and Gynecology guidelines on postpartum diabetes screening (https://www.obgproject.com/2017/06/25/acog-releases-updated-guidance-gestational-diabetes/).

¶¶¶ Additional information about the National Diabetes Prevention Program (https://www.cdc.gov/diabetes/prevention/index.html).
